# Comparison and Research on Diversified Portfolios with Several Entropy Measures Based on Different Psychological States

**DOI:** 10.3390/e22101125

**Published:** 2020-10-04

**Authors:** Xue Deng, Tao Lin, Chuangjie Chen

**Affiliations:** 1 School of Mathematics, South China University of Technology, Guangzhou 510640, China; 201820127121@mail.scut.edu.cn; 2 School of Mathematics, Sun Yat–Sen University, Guangzhou 510275, China; lint_2018@163.com

**Keywords:** portfolio model, different psychological states, possibility theory, various entropy measures

## Abstract

In previous studies, there were few portfolio models involving investors’ psychological states, market ambiguity and entropy. Some entropy can make the model have the effect of diversifying investment, which is very important. This paper mainly studies four kinds of entropy. First, we obtained four definitions of entropy from the literature, and gave the function of fuzzy entropy in different psychological states through strict mathematical proof. Then, we construct a fuzzy portfolio entropy decision model based on the investor’s psychological states, and compared it with the possibilistic mean–variance model. Then we presented a numerical example and compared the five different models established. By comparing the results, we find that: (a) The possibilistic mean–Shannon entropy model solves the problem of the possibility of excessive concentration in the possibilistic mean–variance model, but the dispersion is not enough. Conversely, the possibilistic mean–Yager entropy is over–emphasized due to the definition of its own function, such that it gave an investment pattern of equal weight distribution or approximate average distribution. (b) The results of possibilistic mean–proportional entropy can be said to be the middle status of the portfolios of possibilistic mean–Shannon entropy and possibilistic mean–Yager entropy. This portfolio not only achieves a certain rate of return, but also disperses the risk to some extent. (c) The lines of satisfaction for portfolios derived from different models are approximately U–shaped with the increase in return preference. (d) The possibilistic mean–Shannon entropy model tends to have the highest portfolio satisfaction with the same psychological state of the investor.

## 1. Introduction

Investors and researchers have attached great importance to the allocation of investment portfolios in the securities market. In 1952, Markowitz [1] first proposed the portfolio theory, established the mean–variance (M–V) model, and guided investors to choose the right portfolio under certain indicators and constraints. This theory can be called the beginning of modern portfolio theory. With the study of behavioral finance in the financial field, many researchers began to improve the traditional mean–variance model. Li et al. [2] discussed the issue of portfolio selection in uncertain environments. In order to reflect people’s different attitudes toward risk as the target changes, they considered people’s psychological accounts, introduced uncertain variables to describe securities returns, and considered uncertain portfolio selection and liquidity risk control. In addition, they also considered some important constraints, such as transaction costs, minimum transaction limits, and securities diversification. In their study [3], Wang and Jin showed that investors are more sensitive to losses than gains with respect to the reference point. Therefore, under the framework of prospect theory, they established a multi–stage loss aversion portfolio optimization model based on dynamic loss aversion psychology. In another study [4], Jin and Wang divided the historical data of China’s stock market into three periods: rising, falling and consolidating. They studied the investment performance of dynamic loss aversion portfolio model, static loss aversion portfolio model, M–V model and conditional value at risk (CVaR) model in different periods, and found that the dynamic loss aversion portfolio model is always better than others. Zhan [5] also considered the psychological characteristics of investors’ loss aversion. They constructed a portfolio model based on linear loss aversion and nonlinear loss aversion behavior, and used domestic market data to simulate a static scenario and four dynamic scenarios, which more specifically study the performance of investment performance under different loss aversion scenarios. Li et al. [6] proceeded from the perspective of expectation satisfaction. They thought that people are not pursuing the maximization of rational utility, but maximization of psychological satisfaction. They defined the satisfaction by the difference between the portfolio return and the minimum return of the securities, and finally constructed a fuzzy portfolio model based on the maximum expected satisfaction of the background risk.

On the other hand, considering the ambiguity of the portfolio model in the securities market, Zhou [7] studied the portfolio based on conservative–neutral–radical attitudes. They set the stock return rate as a fuzzy variable. They considered that people with different risk attitudes may have different understandings of the probability of the occurrence of an event. Starting from three different risk attitudes, based on the “Me” defined in the article, they quantified the return and risk levels of the portfolio using the expected and minimum absolute differences of the fuzzy returns. Similar to them, Zhuang [8] studied the influence of three risk attitudes of investors on the portfolio in the paper. The author regarded the rate of return as a trapezoidal fuzzy number, introduced the risk adaptation parameter on the membership function, and used different values of the parameter to correspond to different risk attitudes. By establishing a fuzzy portfolio model, three different risk adaptations were substituted for comparison. Coincidentally, Miao [9] also used the rate of return as a random fuzzy variable in the article, but used the Markov process to predict the return rate of the constructed portfolio.

In addition, whether it is to construct a multi–stage loss aversion portfolio from the psychological behavior of investors, or a model based on fuzzy construction, Chen et al. [10] used Shannon entropy to describe the degree of uncertainty of each rate of return distribution, and then constructed an entropy decision model based on the characteristics of investors’ psychological behavior, which effectively compensates for the shortcomings of traditional securities investment models. Liu et al. [11] introduced the entropy theory and used the maximum entropy distribution to empirically analyze the data. Jiang [12] constructed a portfolio model based on different entropy in the paper, and compared the results. Zhang et al. [13] proposed a mean–semivariance–entropy model for multi–period portfolio selection, and discussed the effectiveness of the model through four indicators of return, risk, transaction cost and portfolio dispersion. Rupak et al. [14] also considered the third–order moment skewness and cross entropy in the model including the mean and variance. Although these models also take entropy, fuzziness or investor attitudes into account, their emphasis is not the same as our model. In this paper, we mainly study and compare the models with different risk attitudes and different entropy, so as to help investors choose the appropriate model according to their own risk attitude and demand.

In order to allow investors to choose models suitable for their risk attitudes, we have established different models based on different entropy, and finally gave the characteristics of the corresponding models. Investors can choose their own model according to the characteristics of the model. As mentioned above, considering that entropy can be used as a new indicator in the portfolio model, we combine the investor’s psychological state, fuzziness and different entropy to construct the model. Then we conduct an empirical analysis of the model and compare the results obtained from the empirical analysis. In the end, we get some useful conclusions about the portfolios with several entropy measures based on different psychological states. The main conclusions are as follows: (i) The possibilistic mean–Shannon entropy model solves the problem of excessive concentrated investment in the possibilistic mean–variance model, but the dispersion is not enough. (ii) The possibilistic mean–Yager entropy over–emphasizes the dispersion due to the definition of its own function, which leads to an investment pattern of equal weight distribution or approximate average distribution. (iii) The results of possibilistic mean–proportional entropy can be said to be the middle status of the portfolios of possibilistic mean–Shannon entropy and possibilistic mean–Yager entropy. This portfolio not only achieves a certain rate of return, but also disperses the risk to some extent.

The rest of the paper is structured as follows: Section 2 shows possibility theory, different psychological states and several entropy measures. Next, we get the function of fuzzy entropy in different psychological states through strict mathematical proof in Section 3. Then, in Section 4, we construct the possibilistic mean–variance model, possibilistic mean–Shannon entropy model, possibilistic mean–fuzzy entropy model, possibilistic mean–Yager entropy model and possibilistic mean–proportional entropy model. Then, in Section 5 we conduct an empirical analysis of the specific examples and get the corresponding results. Finally, we draw some conclusions in Section 6 based on the work done in this article.

## 2. Preliminaries

### 2.1. Possibility Theory

In 1965, Zadeh [15] proposed the concept of fuzzy sets. In the study, he assigned a membership function to each element in the set. The membership function $\mu_{A}(x)$ with range [0,1] is used to indicate the degree to which the element x belongs to the fuzzy set A.

**Definition** **1.** *Assuming that A is a fuzzy number, the γ–level set*${\left[A\right]}^{\gamma }=\left\{x\in R|\mu_{A}(x)\geq \gamma \right\}$*of A can be expressed as*${\left[A\right]}^{\gamma }=\left[a_{1}(\gamma ),a_{2}(\gamma )\right]$*, where*$a_{1}(\gamma )=\min\left\{x\in R|\mu_{A}(x)\geq \gamma \right\}$*,*$a_{2}(\gamma )=\max\left\{x\in R|\mu_{A}(x)\geq \gamma \right\}$*,*γ∈[0,1].

**Definition** **2.** *If* A=(a,b;α,β) *is a fuzzy number, where* $L_{A}$ *and* $R_{A}$ *are continuous functions. Monotonically increase on* [0,1]→[0,1] *, and* $L_{A}(0)=R_{A}(0)=1$ *,* $L_{A}(1)=R_{A}(1)=0$ *, then the membership functions of A are as follows:*

(1) $$\mu_{A}=\{\begin{array}{cc} L_{A}(\frac{a-x}{\alpha }), & a-\alpha \leq x<a;\\ 1, & a\leq x<b;\\ R_{A}(\frac{x-b}{\beta }), & b\leq x<b+\beta ;\\ 0, & \mathrm{other}. \end{array}$$

When $L_{A}$, $R_{A}$ degenerate into a linear function, then A=(a,b;α,β) is called a trapezoidal fuzzy number, whose membership function is:

(2) $$\mu_{A}=\{\begin{array}{cc} 1-(\frac{a-x}{\alpha }), & a-\alpha \leq x<a;\\ 1, & a\leq x<b;\\ 1-(\frac{x-b}{\beta }), & b\leq x<b+\beta ;\\ 0, & \mathrm{other}. \end{array}$$

When $L_{A}$, $R_{A}$ degenerate into a linear function and *a* = *b*, then A=(a,b;α,β) is called a triangular fuzzy number, whose membership function is:

(3) $$\mu_{A}=\{\begin{array}{cc} 1-(\frac{a-x}{\alpha }), & a-\alpha \leq x<a;\\ 1-(\frac{x-b}{\beta }), & b\leq x<b+\beta ;\\ 0, & \mathrm{other}. \end{array}$$

When $L_{A}$, $R_{A}$ degenerate into a linear function and *α* = *β* = 0, *A* is the interval number [*a*, *b*];

When $L_{A}$, $R_{A}$ degenerate into a linear function and *a* = *b*, *α* = *β* = 0, *A* is a real number *a*.

Later, Carlsson and Fuller [16] gave the definition of the upper and lower possibilistic mean in the study, and Zhang and Nie [17] gave the definition of the possibilistic variance and covariance. Since then, more and more researchers have constructed fuzzy portfolio models based on possibilistic mean and possibilistic variance in a fuzzy environment instead of the traditional mean and variance.

Assuming that the *γ*–level set of the fuzzy numbers *A* and *B* are $\left[A]=[a_{1}(\gamma ),a_{2}(\gamma )\right]$ and $\left[B]=[b_{1}(\gamma ),b_{2}(\gamma )],\;\gamma \in [0,1\right]$, respectively, the possibilistic mean given by Carlsson and Fuller and the possibilistic variance and covariance given by Zhang and Nie are defined as follows:

**Definition** **3.** *The possibilistic mean M(A) of the fuzzy number A is defined as:*

(4) $$M(A)=\int_{0}^{1}\gamma [a_{1}(\gamma )+a_{2}(\gamma )]d\gamma .$$

Furthermore, the possibilistic mean of the fuzzy number *A* has the same operational properties as the expectation in probability theory.

**Definition** **4.** *The possibilistic variance* *Var(A)* *of the fuzzy number* *A* *is defined as:*

(5) $$Var(A)=\frac{1}{2}\int_{0}^{1}\gamma {\left[a_{2}(\gamma )-a_{1}(\gamma )\right]}^{2}d\gamma .$$

**Definition** **5.** *The possibilistic covariance* *Cov(A, B)* *of the fuzzy numbers* *A* *and* *B* *is defined as:*

(6) $$Cov(A,B)=\frac{1}{2}\int_{0}^{1}\gamma [a_{2}(\gamma )-a_{1}(\gamma )][b_{2}(\gamma )-b_{1}(\gamma )]d\gamma .$$

Similarly, the possibilistic variance and possibilistic covariance of fuzzy numbers have the same operational properties as the variance and covariance in probability theory.

### 2.2. Different Psychological States

#### 2.2.1. The Investor’s Psychological Characterization Function

When the securities market is observed from the perspective of fuzzy phenomena, the return rate of assets in the securities market is often regarded as a fuzzy number to describe the fuzzy characteristics of the return rate. In this study, it is assumed that the return rate of the i–th assets $r_{i}=(a_{i},b_{i};\alpha_{i},\beta_{i})$ in the securities market is a trapezoidal fuzzy number, where $\left[a_{i},b_{i}\right]$ represents the center interval of the trapezoidal fuzzy number, $\alpha_{i}$ and $\beta_{i}$, respectively, represent the left and right widths which are all greater than zero, i=1,2,⋯n. In order to describe the changes in the investor’s psychological states and the investor’s attitude toward the risk, the risk adaptation parameter *k* (*k* > 0) is introduced into the membership function of $r_{i}$. The investor’s psychological characterization function is as follows:

(7) $$\mu_{r_{i}}(x)=\{\begin{array}{cc} 1-{\left(\frac{a_{i}-x}{\alpha_{i}}\right)}^{k}, & a_{i}-\alpha_{i}\leq x<a_{i};\\ 1, & a_{i}\leq x<b_{i};\\ 1-{\left(\frac{x-b_{i}}{\beta_{i}}\right)}^{k}, & b_{i}\leq x<b_{i}+\beta_{i};\\ 0, & \mathrm{other}. \end{array}$$

The second derivative of Equation (7) is obtained as:

(8) $$\mu_{r_{i}}^{\prime \prime }(x)=\left\{ \begin{array}{c} \begin{array}{cc} -\frac{k(k-1)}{\alpha_{i}^{2}}{\left(\frac{a_{i}-x}{\alpha_{i}}\right)}^{k-2}, & a_{i}-\alpha_{i}\leq x<a_{i},\\ -\frac{k(k-1)}{\beta_{i}^{2}}{\left(\frac{x-b_{i}}{\beta_{i}}\right)}^{k-2}, & b_{i}\leq x<b_{i}+\beta_{i}. \end{array}\\ \;0,\;\;\;\mathrm{other}. \end{array}\right.$$

From Equation (8), it can be seen that when *k* < 1, the investor’s psychological characterization function is concave, indicating that investors are averse to risk; when *k* = 1, the investor’s psychological characterization function is linear, indicating the investors have a neutral attitude towards risk; when *k* > 1, the investor’s psychological characterization function is convex, indicating that the investors have a preference for risk.

#### 2.2.2. Different Psychological States with the Change of *k* Values

After the investor’s psychological characterization function is obtained, the *γ*–level set of the return rate $r_{i}$ of the *i*–th asset can be derived from the Definition 1:

(9) $${\left[r_{i}\right]}^{\gamma }=[r_{i1},r_{i2}]=[a_{i}-{\left(1-\gamma \right)}^{\frac{1}{k}}\alpha_{i},b_{i}+{\left(1-\gamma \right)}^{\frac{1}{k}}\beta_{i}],\gamma \in [0,1].$$

The possibilistic mean and possibilistic variance that can be obtained by combining Equation (9) with Definitions 3 and 4 are as follows:

(10) $$M(r_{i})=\frac{a_{i}+b_{i}}{2}+\frac{k^{2}}{\left(1+k)(1+2k\right)}(\beta_{i}-\alpha_{i}),\forall k>0.$$

(11) $$Var(r_{i})=\frac{{\left(b_{i}-a_{i}\right)}^{2}}{4}+\frac{k^{2}}{4(1+k)(2+k)}{\left(\beta_{i}+\alpha_{i}\right)}^{2}+\frac{k^{2}}{\left(1+k)(1+2k\right)}(b_{i}-a_{i})(\beta_{i}+\alpha_{i}).$$

Combining Equation (9) with Definition 5, we can get the possibilistic covariance of $r_{i}$ and $r_{j},\forall i\neq j$, as shown in Equation (12):

(12) $$\begin{array}{ll} Cov(r_{i},r_{j}) & =\frac{k^{2}}{2(1+k)(1+2k)}[(b_{i}-a_{i})(\beta_{j}+\alpha_{j})+(b_{j}-a_{j})(\beta_{i}+\alpha_{i})]\\ & +\frac{k^{2}}{4(1+k)(2+k)}(\beta_{i}+\alpha_{i})(\beta_{j}+\alpha_{j})+\frac{\left(b_{i}-a_{i})(b_{j}-a_{j}\right)}{4}. \end{array}$$

Therefore, assuming that the investor chooses the asset in *n* and $x_{i}(i=1,2,\cdots ,n)$ represents the investment ratio of the *i*–th asset, the possibilistic mean function of the portfolio strategy is:

(13) $$M(\sum_{i=1}^{n}x_{i}r_{i})=\sum_{i=1}^{n}[\frac{a_{i}+b_{i}}{2}x_{i}+\frac{k^{2}}{\left(1+k)(1+2k\right)}(\beta_{i}-\alpha_{i})x_{i}],\forall k>0.$$

Similarly, the possible variance function for this portfolio strategy is:

(14) $$\begin{array}{ll} Var(\sum_{i=1}^{n}x_{i}r_{i}) & =\sum_{i=1}^{n}[\frac{{\left(b_{i}-a_{i}\right)}^{2}}{4}x_{i}{}^{2}+\frac{k^{2}}{4(1+k)(2+k)}{\left(\beta_{i}+\alpha_{i}\right)}^{2}x_{i}{}^{2}\\ & +\frac{k^{2}}{\left(1+k)(1+2k\right)}(b_{i}-a_{i})(\beta_{i}+\alpha_{i})x_{i}{}^{2}]\\ & +\sum_{i>j=1}^{n}2x_{i}x_{j}\{\frac{\left(b_{i}-a_{i})(b_{j}-a_{j}\right)}{4}+\frac{k^{2}}{4(1+k)(2+k)}(\beta_{i}+\alpha_{i})(\beta_{j}+\alpha_{j})\\ & +\frac{k^{2}}{2(1+k)(1+2k)}[(b_{i}-a_{i})(\beta_{j}+\alpha_{j})+(b_{j}-a_{j})(\beta_{i}+\alpha_{i})]\}. \end{array}$$

Next, we select *k* = 0.5, *k* = 1 and *k* = 2 to represent risk averse investors, risk neutral investors and risk preference investors, respectively. Substituting different *k* values into Equations (13) and (14), respectively, calculate the results. When *k* = 0.5 (i.e., the investor is risk–averse), the corresponding possibilistic mean function and possibilistic variance function are expressed as follows:

(15) $$M(\sum_{i=1}^{n}x_{i}r_{i})=\sum_{i=1}^{n}[\frac{a_{i}+b_{i}}{2}x_{i}+\frac{1}{12}(\beta_{i}-\alpha_{i})x_{i}].$$

(16) $$\begin{array}{ll} Var(\sum_{i=1}^{n}x_{i}r_{i}) & =\sum_{i=1}^{n}[\frac{{\left(b_{i}-a_{i}\right)}^{2}}{4}x_{i}{}^{2}+\frac{1}{60}{\left(\beta_{i}+\alpha_{i}\right)}^{2}x_{i}{}^{2}+\frac{1}{12}(b_{i}-a_{i})(\beta_{i}+\alpha_{i})x_{i}{}^{2}]\\ & +\sum_{i>j=1}^{n}2x_{i}x_{j}\{\frac{\left(b_{i}-a_{i})(b_{j}-a_{j}\right)}{4}+\frac{1}{60}(\beta_{i}+\alpha_{i})(\beta_{j}+\alpha_{j})\\ & +\frac{1}{24}[(b_{i}-a_{i})(\beta_{j}+\alpha_{j})+(b_{j}-a_{j})(\beta_{i}+\alpha_{i})]\}. \end{array}$$

When *k* = 1 (i.e., the investor is risk–neutral), the corresponding possibilistic mean function and possibilistic variance function are expressed as follows:

(17) $$M(\sum_{i=1}^{n}x_{i}r_{i})=\sum_{i=1}^{n}[\frac{a_{i}+b_{i}}{2}x_{i}+\frac{1}{6}(\beta_{i}-\alpha_{i})x_{i}].$$

(18) $$\begin{array}{ll} Var(\sum_{i=1}^{n}x_{i}r_{i}) & =\sum_{i=1}^{n}[\frac{{\left(b_{i}-a_{i}\right)}^{2}}{4}x_{i}{}^{2}+\frac{1}{24}{\left(\beta_{i}+\alpha_{i}\right)}^{2}x_{i}{}^{2}+\frac{1}{6}(b_{i}-a_{i})(\beta_{i}+\alpha_{i})x_{i}{}^{2}]\\ & +\sum_{i>j=1}^{n}2x_{i}x_{j}\{\frac{\left(b_{i}-a_{i})(b_{j}-a_{j}\right)}{4}+\frac{1}{24}(\beta_{i}+\alpha_{i})(\beta_{j}+\alpha_{j})\\ & +\frac{1}{12}[(b_{i}-a_{i})(\beta_{j}+\alpha_{j})+(b_{j}-a_{j})(\beta_{i}+\alpha_{i})]\}. \end{array}$$

When *k* = 2 (i.e., the investor is risk–preferred), the corresponding possibilistic mean function and possibilistic variance function are expressed as follows:

(19) $$M(\sum_{i=1}^{n}x_{i}r_{i})=\sum_{i=1}^{n}[\frac{a_{i}+b_{i}}{2}x_{i}+\frac{4}{15}(\beta_{i}-\alpha_{i})x_{i}].$$

(20) $$\begin{array}{ll} Var(\sum_{i=1}^{n}x_{i}r_{i}) & =\sum_{i=1}^{n}[\frac{{\left(b_{i}-a_{i}\right)}^{2}}{4}x_{i}{}^{2}+\frac{1}{12}{\left(\beta_{i}+\alpha_{i}\right)}^{2}x_{i}{}^{2}+\frac{4}{15}(b_{i}-a_{i})(\beta_{i}+\alpha_{i})x_{i}{}^{2}]\\ & +\sum_{i>j=1}^{n}2x_{i}x_{j}\{\frac{\left(b_{i}-a_{i})(b_{j}-a_{j}\right)}{4}+\frac{1}{12}(\beta_{i}+\alpha_{i})(\beta_{j}+\alpha_{j})\\ & +\frac{2}{15}[(b_{i}-a_{i})(\beta_{j}+\alpha_{j})+(b_{j}-a_{j})(\beta_{i}+\alpha_{i})]\}. \end{array}$$

### 2.3. Several Entropy Measures

#### 2.3.1. Shannon Entropy

Shannon entropy was first proposed by Shannon [18] in 1948. The concept of “Shannon entropy” is now used in many subject areas. The definition given by Shannon is to describe the degree of uncertainty in the value of discrete random variables with *n* results. The entropy value increases as the uncertainty of the random variable increases, and vice versa.

**Definition** **6.** *The definition of Shannon entropy given to characterize its uncertainty is as follows:*

(21) $$S=-k\sum_{i=1}^{n}p_{i}\ln p_{i}.$$

*whereSrepresents a measure of uncertainty,nrepresents a random variable with *n* results, and each result corresponds to a discrete possibility of*$p_{i}(p_{i}>0)$*,*$\sum_{i=1}^{n}p_{i}=1$. *kis a positive constant number depending on the unit of measure, generally takingk= 1.*

#### 2.3.2. Fuzzy Entropy

The concept of fuzzy entropy is an important research topic in fuzzy sets. Compared with general entropy, fuzzy entropy measures the fuzzy uncertainty of a fuzzy set. Luca and Termini [19] first defined non–probabilistic entropy using fuzzy theory. Later, many scholars studied fuzzy entropy and gave their definitions of fuzzy entropy, but these entropy definitions describe the uncertainty caused by language ambiguity rather than information loss. In 2008, Li and Liu [20] proposed a new definition of fuzzy entropy, which describes the uncertainty caused by insufficient information due to the inability to accurately predict the specified parameters. Assuming a fuzzy variable *r*, its corresponding fuzzy entropy is defined as follows:

**Definition** **7.** *The fuzzy entropy of the fuzzy variable r is defined as:*

(22) $$H=-\int_{-\infty }^{\infty }\{\frac{\mu_{r}(x)}{2}\ln\frac{\mu_{r}(x)}{2}+[1-\frac{\mu_{r}(x)}{2}]\ln[1-\frac{\mu_{r}(x)}{2}]\}dr.$$

*where*$\mu_{r}(x)$*represents the membership function corresponding to the fuzzy variabler*.

#### 2.3.3. Yager Entropy

With the study of the concept of entropy, it is hoped that a new kind of entropy can be achieved to retain the properties of the entropy that has been studied before, and to achieve the purpose of simplifying the calculation. In this context, Yager [21] introduced a new information aggregation technique, based on the command weighting operator, in 1988. Later, in 1995, Yager entropy was defined based on the Minkowski distance. Suppose $x=(x_{1},x_{2},\cdots ,x_{n})$ is any point in the Euclidean *n*-dimensional space, and [1/n]=(1/n,1/n,⋯,1/n), *Q(x)* is the Minkowski distance function in *n*-dimensional space to measure the distance between points *x* and [1/n], we can get the expression of *Q(x)* as follows:

(23) $$Q(x)=D(x,\left[\frac{1}{n}\right])={\left(\sum_{i=1}^{n}{\left|x_{i}-\frac{1}{n}\right|}^{z}\right)}^{\frac{1}{z}}.$$

By the geometric properties of the Euclidean space, when *x* = [*I*] (where [*I*] is the unit vector), *Q(x)* can take the maximum value as follows:

(24) $$Q([I])={\left({\left(\frac{n-1}{n}\right)}^{z}+(n-1){\left(\frac{1}{n}\right)}^{z}\right)}^{\frac{1}{z}}=\frac{n-1}{n}{\left(1+\frac{1}{{\left(n-1\right)}^{z-1}}\right)}^{\frac{1}{z}}.$$

**Definition** **8.** *The Yager entropy based on the non–negative nature of entropy is defined as:*

(25) $$H(x)=Q([I])-Q(x)=\frac{n-1}{n}{\left(1+\frac{1}{{\left(n-1\right)}^{z-1}}\right)}^{\frac{1}{z}}-{\left(\sum_{i=1}^{n}{\left|x_{i}-\frac{1}{n}\right|}^{z}\right)}^{\frac{1}{z}}.$$

#### 2.3.4. Proportional Entropy

Most of the existing studies on portfolio and entropy have focused on Shannon entropy and Yager entropy. However, more and more studies have found that Shannon entropy and Yager entropy have some shortcomings based on their own function definitions. For Shannon entropy, the logarithmic function in its definition makes it easy to present a low diversification, which will make it impossible for investors to achieve the purpose of diversifying risks; for Yager entropy, the function definition also makes the obtained solution often show the characteristics of average distribution. However, in actual securities investment, few investors adopt an equal division strategy for asset allocation. Therefore, considering the nature of the two entropies, based on the Minkowski distance, the concept of proportional entropy is proposed. First, a new entropy measure is constructed as follows [12]:

(26) $$Q(x)={\left(\sum_{i=1}^{n-1}{\left|x_{i}-x_{i+1}\right|}^{z}\right)}^{\frac{1}{z}}.$$

It can be seen from its expression that when *x* = [*I*] ([*I*] is a unit vector), *Q(x)* can take the maximum value as follows:

(27) $$Q(x)={\left({\left(0-1\right)}^{z}+{\left(1-0\right)}^{z}\right)}^{\frac{1}{z}}=2^{\frac{1}{z}}.$$

**Definition** **9.** *The definition of proportional entropy is as follows:*

(28) $$H(x)=Q([I])-Q(x)=2^{\frac{1}{z}}-{\left(\sum_{i=1}^{n-1}{\left|x_{i}-x_{i+1}\right|}^{z}\right)}^{\frac{1}{z}}.$$

## 3. Fuzzy Entropy Functions of Investors with Different Psychological States

We substitute the investor psychological characterization function (Equation (7)) into the definition of fuzzy entropy (Equation (22)). For the *i*-th asset, if the fuzzy number of its return rate is $R_{i}=(a_{i},b_{i};\alpha_{i},\beta_{i})$, the fuzzy entropy of the asset is as follows:

(29) $$\begin{array}{ll} H= & -\int_{a_{i}-\alpha_{i}}^{a_{i}}\{\frac{1}{2}\left[1-{\left(\frac{a_{i}-x}{\alpha_{i}}\right)}^{k}\right]\ln\left[\frac{1}{2}-\frac{1}{2}{\left(\frac{a_{i}-x}{\alpha_{i}}\right)}^{k}\right]\\ & +\left[1-\left(\frac{1}{2}-\frac{1}{2}{\left(\frac{a_{i}-x}{\alpha_{i}}\right)}^{k}\right)\right]\ln\left[1-\left(\frac{1}{2}-\frac{1}{2}{\left(\frac{a_{i}-x}{\alpha_{i}}\right)}^{k}\right)\right]\}dx\\ & -\int_{a_{i}}^{b_{i}}\left[\frac{1}{2}\ln\frac{1}{2}+\left(1-\frac{1}{2}\right)\ln\left(1-\frac{1}{2}\right)\right]dx\\ & -\int_{b_{i}}^{b_{i}+\beta_{i}}\{\frac{1}{2}\left[1-{\left(\frac{x-b_{i}}{\beta_{i}}\right)}^{k}\right]\ln\left[\frac{1}{2}-\frac{1}{2}{\left(\frac{x-b_{i}}{\beta_{i}}\right)}^{k}\right]\\ & +\left[1-\left(\frac{1}{2}-\frac{1}{2}{\left(\frac{x-b_{i}}{\beta_{i}}\right)}^{k}\right)\right]\ln\left[1-\left(\frac{1}{2}-\frac{1}{2}{\left(\frac{x-b_{i}}{\beta_{i}}\right)}^{k}\right)\right]\}dx. \end{array}$$

The three integrals in Formula (29) are abbreviated as *D*, *E*, and *F*, respectively, and the calculations for *D*, *E*, and *F* are as follows:

(30) $$D=-\frac{2\alpha_{i}}{k}\int_{0}^{\frac{1}{2}}[y\ln y+(1-y)\ln(1-y)]{\left(1-2y\right)}^{\frac{1-k}{k}}dy.$$

where $\frac{1}{2}\left[1-{\left(\frac{a_{i}-x}{\alpha_{i}}\right)}^{k}\right]=y$, that is, $x=a_{i}-\alpha_{i}{\left(1-2y\right)}^{\frac{1}{k}}$, and Formula (30) is obtained from D in Formula (29). Similarly, we have:

(31) $$E=\int_{a_{i}}^{b_{i}}\ln2dx=(b_{i}-a_{i})\ln2.$$

(32) $$F=-\frac{2\beta_{i}}{k}\int_{0}^{\frac{1}{2}}[y\ln y+(1-y)\ln(1-y)]{\left(1-2y\right)}^{\frac{1-k}{k}}dy.$$

where $\frac{1}{2}\left[1-{\left(\frac{x-b_{i}}{\beta_{i}}\right)}^{k}\right]=y$, that is, $x=\beta_{i}{\left(1-2y\right)}^{\frac{1}{k}}+b_{i}$, and Formula (32) can be obtained from *F* in Formula (29).

When *k* = 0.5 (i.e., the investor is risk–adverse), Formula (30) can be written as follows:

(33) D=−4αi∫012[ylny+(1−y)ln(1−y)](1−2y)dy=−4αi{(y−y2)[ylny+(1−y)ln(1−y)]|012−∫012(y−y2)[lny−ln(1−y)]dy}= αiln2+4αi∫012(y−y2)[lny−ln(1−y)]dy=αiln2+4αi{(y22−y33)[lny−ln(1−y)]−∫012(y22−y33)1y(1−y)dy}=αiln2−4αi∫0123y−2y26(1−y)dy=αiln23+αi6.

Observing Formulas (30) and (32), we can see that there is only a coefficient difference between the two integrals, and the integral part is the same. Therefore, under this condition, Formula (32) can be written as:

(34) $$F=\frac{\beta_{i}\ln2}{3}+\frac{\beta_{i}}{6}.$$

Combining Formulas (31), (33), and (34), we can get Theorem 1.

**Theorem** **1.** *When k = 0.5 (that is, investors are risk–averse), the fuzzy entropy corresponding to the portfolio is as follows:*

(35) $$H_{FE}(\sum_{i=1}^{n}x_{i}r_{i})=\sum_{i=1}^{n}x_{i}[(\frac{\ln2}{3}+\frac{1}{6})(\alpha_{i}+\beta_{i})+(b_{i}-a_{i})\ln2].$$

When *k* = 1 (i.e., the investor is risk–neutral), Formula (30) has the following specific form:

(36) D=−2αi∫012[ylny+(1−y)ln(1−y)]dy=−2αi∫012ylnydy−2αi∫012(1−y)ln(1−y)dy=−2αi(y22lny|012−∫012y2dy)−2αi[(y−y22)ln(1−y)|012−∫0122y−y22(1−y)dy]=αiln2+αi8−αi∫0122y−y21−ydy=αiln2+αi8+3αi8−αiln2=αi2.

Similarly, Formula (32) can be simplified as:

(37) $$F=\frac{\beta_{i}}{2}.$$

Combining Formulas (31), (36), and (37), we can get Theorem 2.

**Theorem** **2.** *When k = 1 (that is, the investor is risk–neutral), the fuzzy entropy corresponding to the portfolio is as follows:*

(38) $$H_{FE}(\sum_{i=1}^{n}x_{i}r_{i})=\sum_{i=1}^{n}x_{i}[\frac{1}{2}(\alpha_{i}+\beta_{i})+(b_{i}-a_{i})\ln2].$$

Notice that when the investor is in this psychological state, the fuzzy entropy corresponding to the investor’s psychological state is degenerated to the fuzzy entropy of the membership function that is substituted into the ordinary trapezoidal fuzzy number.

When *k* = 2 (i.e., the investor is risk–preferred), Formula (30) is specifically shown as Formula (39). To be convenient, the two integral terms in Formula (39) are, respectively, recorded as *I* and *J*; their calculations are shown in Formulas (40) and (41):

(39) D=−αi∫012[ylny+(1−y)ln(1−y)](1−2y)−12dy=−αi{−(1−2y)12[ylny+(1−y)ln(1−y)]|012+∫012(1−2y)12[lny−ln(1−y)]dy}=−αi∫012(1−2y)[lny−ln(1−y)]dy=−αi∫012(1−2y)lnydy+αi∫012(1−2y)ln(1−y)dy.

(40) $$\begin{array}{ll} I & =-\frac{\alpha_{i}}{2}\int_{0}^{1}\sqrt{z}\left[\ln(1-z)-\ln2\right]dz\\ & =-\frac{\alpha_{i}}{2}\int_{0}^{1}\sqrt{z}\ln(1-z)dz+\frac{\alpha_{i}\ln2}{2}\int_{0}^{1}dz\\ & =-\frac{\alpha_{i}}{2}\times \left(\frac{4}{3}\ln2-\frac{16}{9}\right)+\frac{\alpha_{i}\ln2}{2}\\ & =\frac{8\alpha_{i}}{9}-\frac{\alpha_{i}\ln2}{6}. \end{array}$$

In the process of calculating the integral *I*, let 1−2y=z, that is, $y=\frac{1}{2}-\frac{z}{2}$, and we obtain the results. Similarly, in the calculation of *J*, we let $\sqrt{1-2y}=z$, that is, $y=\frac{1}{2}-\frac{z^{2}}{2}$.

(41) J=αi[−13(1−2y)32ln(1−y)|012−13∫012(1−2y)3211−ydy]=−αi3∫012(1−2y)321−ydy=−2αi3∫01z4z2+1dz=−2αi3∫01(z2−1)dz−2αi3∫011z2+1dz=−2αi3×(−23)−2αi3×π4=4αi9−παi6.

Substituting the results obtained by Formulas (40) and (41) into Formula (39), we have:

(42) $$D=\frac{8\alpha_{i}}{9}-\frac{\alpha_{i}\ln2}{6}+\frac{4\alpha_{i}}{9}-\frac{\pi \alpha_{i}}{6}=\alpha_{i}\left(\frac{4}{3}-\frac{\ln2}{6}-\frac{\pi }{6}\right).$$

Similarly, when *k* = 2 (that is, the investor is risk–preferred), Formula (32) is specified as:

(43) $$F=\beta_{i}\left(\frac{4}{3}-\frac{\ln2}{6}-\frac{\pi }{6}\right).$$

Combining Formulas (31), (42), and (43), we can get Theorem 3.

**Theorem** **3.** *When k = 2 (i.e., the investor is risk–preferred), the fuzzy entropy corresponding to the portfolio is as follows:*

(44) $$H_{FE}(\sum_{i=1}^{n}x_{i}r_{i})=\sum_{i=1}^{n}x_{i}[(\frac{8-\ln2-\pi }{6})(\alpha_{i}+\beta_{i})+(b_{i}-a_{i})\ln2].$$

## 4. Models Based on Different Investor Psychological States

### 4.1. Possibilistic Mean–Variance Model

Before establishing the possibilistic mean–Shannon entropy model, based on the traditional mean–variance model proposed by Markowitz, we construct a possibilistic mean–variance model based on trapezoidal fuzzy numbers. The goal of the model is to maximize the benefits and reduce the risks as much as possible. Therefore, the model built is as follows:

(45) $$\left\{ \begin{array}{ll} \max & M(\sum_{i=1}^{n}x_{i}r_{i}),\\ \min & Var(\sum_{i=1}^{n}x_{i}r_{i}),\\ s.t. & \sum_{i=1}^{n}x_{i}\leq 1,\\ & l_{i}\leq x_{i}\leq u_{i},\forall i=1,2,\cdots ,n. \end{array}\right.$$

In Model (45), the possibilistic mean function measures the return, the possibilistic variance function measures the risk, $\sum_{i=1}^{n}x_{i}\leq 1$ means that only a part of the investable amount is allowed to be traded, and $u_{i}$ and $l_{i}$ are the upper and lower limits of the investment ratio, respectively.

Let $\varphi_{1}(x)$ and $\varphi_{2}(x)$ be the possibilistic mean function and the possibilistic variance function after normalization, as follows:

(46) $$\varphi_{1}(x)=\frac{M(x)-M{\left(x\right)}_{\min}}{M{\left(x\right)}_{\max}-M{\left(x\right)}_{\min}};$$

(47) $$\varphi_{2}(x)=\frac{Var{\left(x\right)}_{\max}-Var(x)}{Var{\left(x\right)}_{\max}-Var{\left(x\right)}_{\min}}.$$

where M(x) represents the possibilistic mean function in Model (45), $M{\left(x\right)}_{\max}$ and $M{\left(x\right)}_{\min}$, respectively, represent the maximum and minimum values obtained when there is only the possibilistic mean as target under the same constraints; Var(x) represents the possibilistic variance function in Model (45), $Var{\left(x\right)}_{\max}$ and $Var{\left(x\right)}_{\min}$, respectively, represent the maximum and minimum values obtained when there is only the possibilistic variance as the target.

We normalize $M(\sum_{i=1}^{n}x_{i}r_{i})$ and $Var(\sum_{i=1}^{n}x_{i}r_{i})$ in Model (45), and combine the two targets to establish satisfaction function $A_{1}(x)$, as shown in (48):

(48) $$\left\{ \begin{array}{ll} \max & A_{1}(x)=\omega_{1}\varphi_{1}(x)+\omega_{2}\varphi_{2}(x),\\ s.t. & \sum_{i=1}^{n}x_{i}\leq 1,\\ & l_{i}\leq x_{i}\leq u_{i},\forall i=1,2,\cdots ,n. \end{array}\right.$$

Here, $\omega_{1}$ and $\omega_{2}$ are the weights indicating the propensity of investors to show the benefits and risks of the portfolio in a particular psychological state, $\omega_{1}+\omega_{2}=1$. When $\omega_{1}$ is larger, it means that the investor is more inclined to obtain interest; when $\omega_{2}$ is larger, it indicates that the investor is more inclined to reduce the risk.

### 4.2. Possibilistic Mean–Shannon Entropy Model

Based on the traditional mean–variance model, at this time, we replace the mean with the possibilistic mean function and measure the risk with the Shannon entropy function as a new indicator. According to the knowledge of securities investment, when the investment is more diversified, the risk of investment will decline. From this perspective, the possibilistic mean–Shannon entropy model is constructed based on the investment dispersion reflected by the Shannon entropy function. The model is as follows:

(49) $$\left\{ \begin{array}{ll} \max & M(\sum_{i=1}^{n}x_{i}r_{i}),\\ \max & H_{SE}=-\sum_{i=1}^{n}x_{i}\ln x_{i},\\ s.t. & \sum_{i=1}^{n}x_{i}\leq 1,\\ & l_{i}\leq x_{i}\leq u_{i},\forall i=1,2,\cdots ,n. \end{array}\right.$$

In this model, $H_{SE}$ represents the index that measures the dispersion of investment ratio defined by the Shannon entropy function, and the meanings of the remaining functions and parameters are the same as those in Model (45).

Similar to Model (45), we normalize the objective function in the above model. The normalization method is similar to Equation (46). After the weighted combination of the normalized functions is obtained, the satisfaction function is constructed. The new model obtained is as follows:

(50) $$\left\{ \begin{array}{ll} \max & A_{2}(x)=\omega_{3}\varphi_{3}(x)+\omega_{4}\varphi_{4}(x),\\ s.t. & \sum_{i=1}^{n}x_{i}\leq 1,\\ & l_{i}\leq x_{i}\leq u_{i},\forall i=1,2,\cdots ,n. \end{array}\right.$$

The meanings of the functions and parameters are the same as in Model (48). When $\omega_{1}$ is larger, it means that the investor is more inclined to obtain interest; when $\omega_{2}$ is larger, it indicates that the investor is more inclined to diversification.

### 4.3. Possibilistic Mean–Fuzzy Entropy Model

In the previous section, we used Shannon entropy to measure the dispersion of a portfolio. The risk does not, however, have to be portrayed from this perspective. It can also be similar to the possibilistic variance in Section 4.1, by directly measuring the degree of fluctuation of the rate of return to characterize the risk. In this section, we will start with the return rate on assets as a fuzzy variable, and calculate the degree of fluctuation of the return rate on assets in the fuzzy environment through fuzzy entropy, and then quantify the risk. Therefore, the fuzzy entropy function in this section is a minimizing objective function. The smaller the value, the more stable the return rate of its corresponding portfolio, and vice versa.

Based on the possibilistic mean–variance Model (45) mentioned in Section 4.1 of this paper, we still retain the possibilistic mean function to reflect the return rate of the portfolio, and then replace the possibilistic variance function with the fuzzy entropy function. The goal of the model is to make the possibilistic mean function as large as possible and the fuzzy entropy function as small as possible. Therefore, the model built is as follows:

(51) $$\left\{ \begin{array}{ll} \max & M(\sum_{i=1}^{n}x_{i}r_{i}),\\ \min & H_{FE}(\sum_{i=1}^{n}x_{i}r_{i}),\\ s.t. & \sum_{i=1}^{n}x_{i}\leq 1,\\ & l_{i}\leq x_{i}\leq u_{i},\forall i=1,2,\cdots ,n. \end{array}\right.$$

where $H_{FE}(\sum_{i=1}^{n}x_{i}r_{i})$ represents the fuzzy entropy function, and the meanings of the remaining functions and parameters are the same as in Model (45).

Similar to Model (45), we normalize the objective functions in the above model, and the normalization of the possibilistic mean function is the same as Equation (46). The normalization of the fuzzy entropy function is in the same form as Equation (47). The normalized functions are weighted and combined to obtain the satisfaction function $A_{3}(x)$. The new model is as follows:

(52) $$\left\{ \begin{array}{ll} \max & A_{3}(x)=\omega_{5}\varphi_{5}(x)+\omega_{6}\varphi_{6}(x),\\ s.t. & \sum_{i=1}^{n}x_{i}\leq 1,\\ & l_{i}\leq x_{i}\leq u_{i},\forall i=1,2,\cdots ,n. \end{array}\right.$$

The meanings of the functions and parameters are similar to Model (48).

### 4.4. Possibilistic Mean–Yager Entropy Model

In order to find different entropy to measure the portfolio dispersion and achieve the purpose of further improving the portfolio results obtained by Model (49), we still retain the possibilistic mean function to reflect the return of the portfolio, and replace Shannon entropy function with Yager entropy function to quantify the dispersion of the portfolio. The goal of the model is to maximize the possibilistic mean function and the Yager entropy (i.e., the investment is as diversified as possible). Therefore, the model is as follows:

(53) $$\left\{ \begin{array}{ll} \max & M(\sum_{i=1}^{n}x_{i}r_{i}),\\ \max & H_{YE}=\frac{n-1}{n}{\left(1+\frac{1}{{\left(n-1\right)}^{z-1}}\right)}^{\frac{1}{z}}-{\left(\sum_{i=1}^{n}{\left|x_{i}-\frac{1}{n}\right|}^{z}\right)}^{\frac{1}{z}},\\ s.t. & \sum_{i=1}^{n}x_{i}\leq 1,\\ & l_{i}\leq x_{i}\leq u_{i},\forall i=1,2,\cdots ,n. \end{array}\right.$$

In Model (53), *H_YE_* represents the Yager entropy function. In this paper, we consider the following three cases: z = 1, z = 2, z is infinity. Their corresponding Yager entropy functions are shown in Formulas (54) to (56), respectively.

(54) $$H_{YE(z=1)}=\frac{2(n-1)}{n}-\sum_{i=1}^{n}\left|x_{i}-\frac{1}{n}\right|.$$

(55) $$H_{YE(z=2)}=\sqrt{1-\frac{1}{n}}-\sqrt{\sum_{i=1}^{n}{\left(x_{i}-\frac{1}{n}\right)}^{2}}.$$

(56) $$H_{YE(z\to \infty )}=1-Max[x_{i}].$$

The rest of the functions and parameters have the same meaning as in Model (49).

Similar to Model (45), we normalize the objective functions in the above model. The normalization of the possibilistic mean function and the Yager entropy function is in the same form as Equation (46). After that, we weight and combine the normalized functions to obtain the satisfaction function $A_{4}(x)$. The new model obtained is as follows:

(57) $$\left\{ \begin{array}{ll} \max & A_{4}(x)=\omega_{7}\varphi_{7}(x)+\omega_{8}\varphi_{8}(x),\\ s.t. & \sum_{i=1}^{n}x_{i}\leq 1,\\ & l_{i}\leq x_{i}\leq u_{i},\forall i=1,2,\cdots ,n. \end{array}\right.$$

The meanings of the functions and parameters are similar to Model (48).

### 4.5. Possibilistic Mean–Proportional Entropy Model

We further check the possibilistic mean–Yager entropy Model (53) mentioned in Section 4.4, and still retain the possibilistic mean function to reflect the return of the portfolio. The proportional entropy function is used instead of the Yager entropy function to quantify the dispersion of the portfolio. The goal of the model is to make the possibilistic mean function as large as possible, and the proportional entropy function as large as possible (i.e., the investment is as diversified as possible). Therefore, the model is built as follows:

(58) $$\left\{ \begin{array}{ll} \max & M(\sum_{i=1}^{n}x_{i}r_{i}),\\ \max & H_{PE}=2^{\frac{1}{z}}-{\left(\sum_{i=1}^{n-1}{\left|x_{i}-x_{i+1}\right|}^{z}\right)}^{\frac{1}{z}},\\ s.t. & \sum_{i=1}^{n}x_{i}\leq 1,\\ & l_{i}\leq x_{i}\leq u_{i},\forall i=1,2,\cdots ,n. \end{array}\right.$$

In Model (58), *H_PE_* represents the proportional entropy function. In this paper, we consider the following three cases: *z* = 1, *z* = 2, *z* is infinity. Their corresponding proportional entropy functions are shown in Formulas (59) to (61), respectively.

(59) $$H_{PE(z=1)}=2-\sum_{i=1}^{n-1}\left|x_{i}-x_{i+1}\right|.$$

(60) $$H_{PE(z=2)}=\sqrt{2}-\sqrt{\sum_{i=1}^{n-1}{\left|x_{i}-x_{i+1}\right|}^{2}}.$$

(61) $$H_{PE(z\to \infty )}=1-\underset{i=1}{Max}\left|x_{i}-x_{i+1}\right|.$$

Similar to Model (45), we normalize the objective functions in the above model. The normalization of the possibilistic mean function and the proportional entropy function is in the same form as Equation (46). After that, we weight and combine the normalized functions to obtain the satisfaction function $A_{5}(x)$. The new model obtained is as follows:

(62) $$\left\{ \begin{array}{ll} \max & A_{5}(x)=\omega_{9}\varphi_{9}(x)+\omega_{10}\varphi_{10}(x),\\ s.t. & \sum_{i=1}^{n}x_{i}\leq 1,\\ & l_{i}\leq x_{i}\leq u_{i},\forall i=1,2,\cdots ,n. \end{array}\right.$$

The meanings of the functions and parameters are similar to Model (48).

## 5. A Numerical Example

Considering such factors as stock scale, liquidity and profitability, we select 10 stocks of different industries from the Shanghai and Shenzhen 300 Index as the research object for the next empirical analysis. The selected stock codes are: 000651, 600340, 600887, 000858, 600196, 600297, 600977, 300015, 600339, and 000166. We select the weekly closing prices for the two years from 26 January 2017 to 22 February 2019 to calculate the weekly return rate from February 2017 to February 2019. After that, we select the 40th percentile and the 60th percentile of the weekly return rate as the left and right endpoints of the center interval of the trapezoidal fuzzy number, and the 5th and the 95th percentile as the parameters a−α and b+β to construct the trapezoidal fuzzy variable as the return rate of stocks. The calculation formula of the weekly return rate $r_{it}$ of the *t*-week of the *i*-th stock is as follows:

(63) $$r_{it}=\frac{p_{i,t}-p_{i,t-1}}{p_{i,t-1}}.$$

where, $p_{i,t}$ represents the weekly closing price of the *t*-week of the *i*-th stock.

The weekly return rates of each stock as a trapezoidal fuzzy number $r_{i}=(a_{i},b_{i};\alpha_{i},\beta_{i})$ corresponding to $a_{i}$, $b_{i}$, $\alpha_{i}$ and $\beta_{i}$ are shown in Table 1:

In this paper, we set the minimum investment ratio for each stock to be 0.05, and the upper limit is 0.5.

In addition, we find that the possibilistic variance and fuzzy entropy represent similar meanings. In order to better compare the possibilistic mean–fuzzy entropy model and the possibilistic mean–variance model, the return reflected by the possibilistic mean is briefly written as *Earnings*, the risks reflected by fuzzy entropy and the square root of the possibilistic variance are briefly written as *Risk*. Then the Sharpe Ratio (SR) is *SR* = *Earnings*/*Risk*. When the *SR* is larger, it means that the return corresponding to the unit risk is larger, and the performance of the portfolio is better, and vice versa.

### 5.1. Performance of the Same Model under Different Psychological States

To observe the changes in investor satisfaction with the propensity of the two indicators of the proposed models, the value of $\omega_{1}$ was changed from 0.1 to 0.9 in each model, with an increment of 0.1 each time. We used Matlab to solve the model and get the satisfaction of investors with different psychological states in the same model for the portfolio, which are shown in Table 2, Table 3, Table 4, Table 5 and Table 6. For convenience sake, the possibilistic mean–variance (M–V) model, the possibilistic mean–Shannon entropy (M–SE) model, the possibilistic mean–fuzzy entropy (M–FE) model, the possibilistic mean–Yager entropy (M–YE) model and the possibilistic mean–proportional entropy (M–PE) model are, respectively, abbreviated as M–V model, M–SE model, M–FE model, M–YE model and M–PE model.

**Figure 1:**
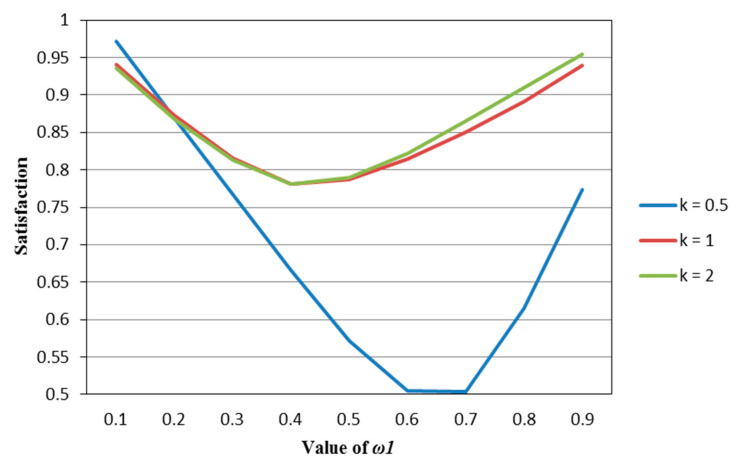
The satisfaction of the M–V model (Model (45)) in different psychological states.

**Figure 2:**
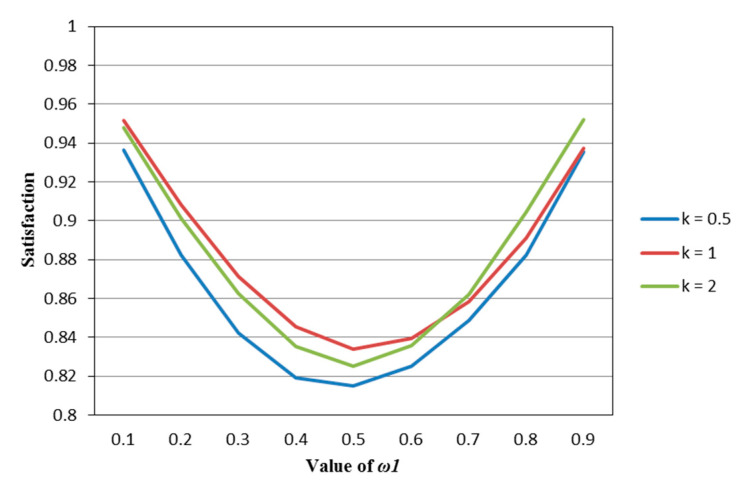
The satisfaction of the M–SE model (Model (49)) in different psychological states.

**Figure 3:**
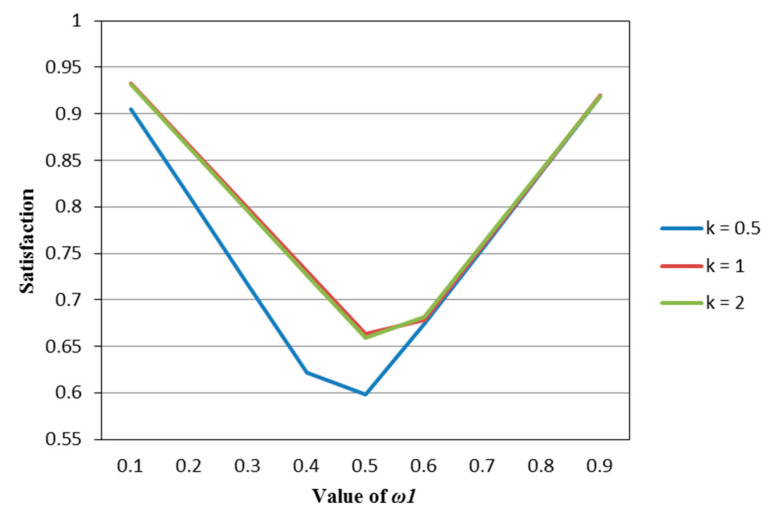
The satisfaction of the M–FE model (Model (51)) in different psychological states.

**Figure 4:**
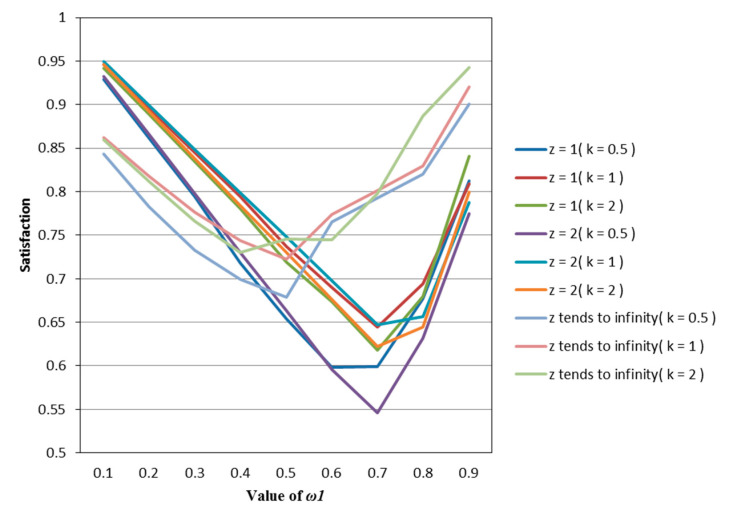
The satisfaction of the M–YE model (Model (53)) in different psychological states.

**Figure 5:**
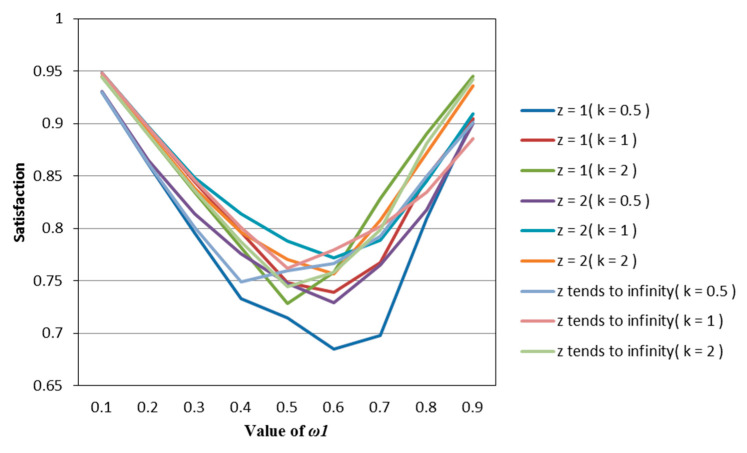
The satisfaction of the M–PE model (Model (58)) in different psychological states.

According to Table 2, Table 3, Table 4, Table 5 and Table 6, we draw Figure 1, Figure 2, Figure 3, Figure 4 and Figure 5.

It can be seen from Figure 1, Figure 2, Figure 3, Figure 4 and Figure 5 that under different psychological states, the satisfaction of all models is approximately U–shaped. Considering that the two indicators involved in all the models are mutually conflicting, and $\omega_{1}$ represents the weight of the possibilistic mean as the first indicator, as $\omega_{1}$ increases, it means that the other indicator is decreasing. The interaction between the two indicators leads to a U–shaped distribution when the values of satisfaction are connected. In addition, it can be seen that when the models are the same, the satisfaction of *k* = 0.5 (i.e., the investor is risk–averse) is often the lowest; the satisfaction of *k* = 1 (that is, the investor is risk–neutral) is higher than the satisfaction of *k* = 2 (i.e., the investor is risk–preferred) when $\omega_{1}$ is small, and is lower than the satisfaction of *k* = 2 when $\omega_{1}$ is large. The reason is that risk–preferred investors will pay more attention to the benefits rather than reduce or spread the risks, so when $\omega_{1}$ is larger, their satisfaction will be greater.

### 5.2. Performance of Different Models in the Same Psychological State

Similar to Section 5.1, when $\omega_{1}$ changes as above, we use Matlab to measure the satisfaction of the investors with the same psychological state for different models, as shown in Table 7, Table 8 and Table 9.

**Figure 6:**
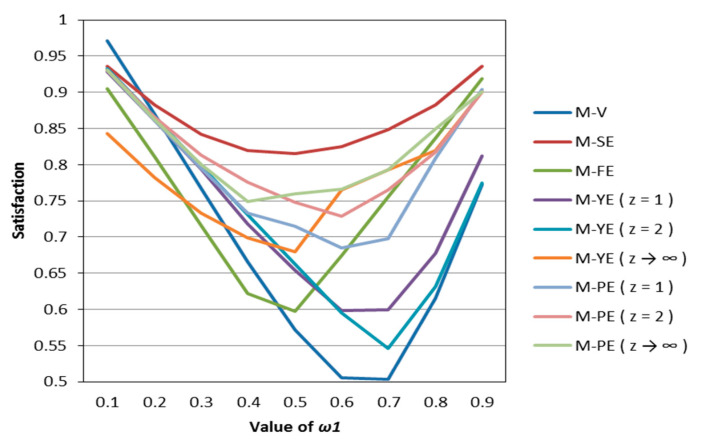
The satisfaction of different models when *k* = 0.5.

**Figure 7:**
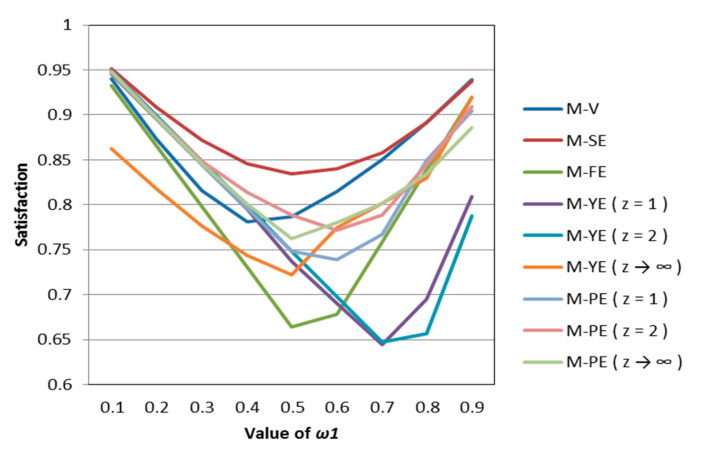
The satisfaction of different models when *k* = 1.

**Figure 8:**
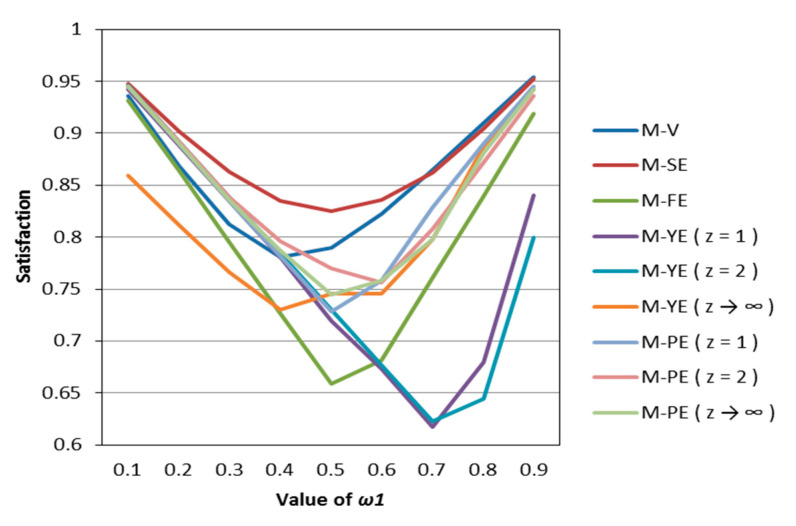
The satisfaction of different models when *k* = 2.

According to Table 7, Table 8 and Table 9, we draw Figure 6, Figure 7 and Figure 8. Observing Figure 6, Figure 7 and Figure 8, we can see that when in the same mental state, the satisfaction of the portfolio obtained by the possibilistic mean–Yager entropy (*z* = 1) model, possibilistic mean–Yager entropy (*z* = 2) model and possibilistic mean–fuzzy entropy are always lower than others. From the investment ratio of each stock in the obtained portfolio, it can be found that the proportion of investment obtained by these models excessively emphasizes diversification. They provide a portfolio pattern of approximating equal investment or near–average allocation. For this reason, the investor satisfaction of these models will be lower than other models. In addition, regardless of the psychological state of the investor, the portfolio derived from the possibilistic mean–Shannon entropy model has a high investor satisfaction.

### 5.3. Portfolio Performance of Each Model

To do further analysis, when the value of $\omega_{1}$ is taken as 0.5 (i.e., the investor keeps neutral between return and risk), we use Matlab to solve the models, and evaluate the performance of each model. The results are shown in Table 10, Table 11 and Table 12.

First, we compare the possibilistic mean–variance model and the possibilistic mean–Shannon entropy model. We find that the possibilistic mean–Shannon entropy model does a better job in decentralized investment. This portfolio solves the problem that the investment of the possibilistic mean–variance model is too concentrated, thus satisfying the investor’s expectations and effectively spreading the investment risk.

Since both the possibilistic variance and the fuzzy entropy are indicators of the degree of fluctuation in the weekly return rate of the asset, therefore, we compare the possibilistic mean–variance model and the possibilistic mean–fuzzy entropy model. We find that when solving Model (51), because of the pursuit of low entropy, there is a portfolio of equal weight and low investment ratio. After comparison, we also find that using the concept of fuzzy entropy to describe the fluctuation of securities return rates is not superior to the portfolio obtained by using the possibilistic variance to characterize the fluctuation of securities return rates.

In order to improve the possibilistic mean–Shannon entropy model, we empirically analyze the possibilistic mean–Yager entropy model (*z* = 1, *z* = 2, and *z* is infinity). We find that if we improve the diversification of the portfolio and maintain a high level of satisfaction, choosing the possibilistic mean–Yager entropy model with the condition that *z* is infinity would be more desirable. However, when *z* = 1 and *z* = 2, the Yager entropy function emphasizes the proximity of points in space and [1/n]=(1/n,1/n,⋯,1/n). Because of this definition, the portfolio model based on the concept of Yager entropy tends to make the portfolio produce the phenomenon of complete even distribution in Table 2, that is, the portfolio over–emphasizes the dispersion of assets.

In order to solve the over–average phenomenon that occurs in the possibilistic mean–Yager entropy model, we introduce the concept of proportional entropy. Finally, we empirically analyze the possibilistic mean–proportional entropy model (*z* = 1, *z* = 2, and *z* is infinity) and find that the results of the model can be said to be the middle status of the portfolio of possibilistic mean–Shannon entropy and possibilistic mean–Yager entropy. This portfolio not only achieves a certain rate of return, but also disperses the risk to some extent.

## 6. Conclusions

Entropy is a powerful tool for diversifying investments. In this paper, we consider different psychological states of investors in a fuzzy environment and establish several models based on four different entropies. In the end, we found that portfolio models based on different entropy have different characteristics. The possibilistic mean–Shannon entropy model solves the problem of the excessive concentrated investment in the possibilistic mean–variance model, but the dispersion is not enough. The possibilistic mean–Yager entropy model over–emphasizes the dispersion due to the definition of its own function, so it provides an investment pattern of equal weight distribution or approximate average distribution. The results of possibilistic mean–proportional entropy can be said to be the middle status of the portfolios of possibilistic mean–Shannon entropy and possibilistic mean–Yager entropy. This portfolio not only achieves a certain rate of return, but also disperses the risk to some extent.

In the future, we may do further study to characterize the investor’s psychological states through other functions, and search for the more realistic psychological state function. In addition, for the use of the function that characterizes the investor’s psychological states, in this paper, we only use *k* = 0.5, *k* = 1 and *k* = 2 for research. If we divide the investor’s risk attitude more specifically, we need to choose another risk adaptation value to express it. Similarly, the entropy we selected in this study is Shannon entropy, fuzzy entropy, Yager entropy and proportional entropy, but it does not mean that the portfolio model can only be established from these four entropies. In future research, we can also construct or use other entropies for empirical analysis.

## Figures and Tables

**Table 1:** The $a_{i}$, $b_{i}$, $\alpha_{i}$ and $\beta_{i}$ of trapezoidal fuzzy numbers corresponding to each stock.

Stock Codes	$a_{i}$	$b_{i}$	$\alpha_{i}$	$\beta_{i}$
000651	−0.19%	1.53%	6.50%	7.89%
600340	−0.70%	1.26%	8.96%	7.66%
600887	−0.89%	1.83%	6.16%	5.45%
000858	−0.29%	2.11%	7.86%	6.61%
600196	−0.47%	1.37%	6.66%	6.55%
600297	−0.95%	1.05%	8.44%	7.24%
600977	−0.95%	0.80%	6.41%	5.90%
300015	−0.09%	1.94%	6.05%	6.46%
600339	−1.40%	0.34%	7.91%	8.00%
000166	−0.81%	0.41%	4.01%	4.32%

**Table 2:** The satisfaction of the M–V model (Model (45)) in different psychological states.

	$\omega_{1}$	0.1	0.2	0.3	0.4	0.5	0.6	0.7	0.8	0.9
*k*										
0.5		0.9712	0.8693	0.7674	0.6659	0.5719	0.5050	0.6154	0.6154	0.7731
1		0.9401	0.8727	0.8153	0.7813	0.7871	0.8148	0.8504	0.8918	0.9393
2		0.9358	0.8682	0.8127	0.7809	0.7895	0.8221	0.8646	0.9095	0.9544

**Table 3:** The satisfaction of the M–SE model (Model (49)) in different psychological states.

	$\omega_{1}\;$	0.1	0.2	0.3	0.4	0.5	0.6	0.7	0.8	0.9
*k*										
0.5		0.9365	0.8826	0.8422	0.8194	0.8153	0.8250	0.8487	0.8826	0.9356
1		0.9516	0.9080	0.8715	0.8453	0.8340	0.8397	0.8583	0.8912	0.9373
2		0.9481	0.9016	0.8626	0.8352	0.8253	0.8357	0.8620	0.9046	0.9520

**Table 4:** The satisfaction of the M–FE model (Model (51)) in different psychological states.

	$\omega_{1}$	0.1	0.2	0.3	0.4	0.5	0.6	0.7	0.8	0.9
*k*										
0.5		0.9055	0.8109	0.7163	0.6218	0.5979	0.6746	0.7556	0.8372	0.9191
1		0.9328	0.8656	0.7983	0.7310	0.6638	0.6784	0.7588	0.8392	0.9196
2		0.9318	0.8635	0.7952	0.7270	0.6587	0.6811	0.7604	0.8397	0.9190

**Table 5:** The satisfaction of the M–YE model (Model (53)) in different psychological states.

	$\omega_{1}$	0.1	0.2	0.3	0.4	0.5	0.6	0.7	0.8	0.9
	
*z* = 1 (*k* = 0.5)		0.9288	0.8614	0.7941	0.7185	0.6542	0.5982	0.5991	0.6773	0.8121
*z* = 1 (*k* = 1)		0.9458	0.8954	0.8451	0.7948	0.7373	0.6902	0.6444	0.6946	0.8091
*z* = 1 (*k* = 2)		0.9422	0.8884	0.8345	0.7807	0.7189	0.6732	0.6177	0.6800	0.8404
*z* = 2 (*k* = 0.5)		0.9326	0.8652	0.7978	0.7304	0.6631	0.5957	0.5457	0.6314	0.7750
*z* = 2 (*k* = 1)		0.9496	0.8992	0.8489	0.7985	0.7481	0.6977	0.6474	0.6567	0.7874
*z* = 2 (*k* = 2)		0.9461	0.8922	0.8383	0.7844	0.7304	0.6765	0.6226	0.6442	0.7994
*z* → ∞ (*k* = 0.5)		0.8433	0.7821	0.7325	0.6993	0.6792	0.7653	0.7925	0.8200	0.9009
*z* → ∞ (*k* = 1)		0.8625	0.8178	0.7765	0.7440	0.7225	0.7742	0.8014	0.8299	0.9200
*z* → ∞ (*k* = 2)		0.8596	0.8115	0.7665	0.7304	0.7453	0.7451	0.7981	0.8870	0.9424

**Table 6:** The satisfaction of the M–PE model (Model (58)) in different psychological states.

	$\omega_{1}$	0.1	0.2	0.3	0.4	0.5	0.6	0.7	0.8	0.9
	
*z* = 1 (*k* = 0.5)		0.9302	0.8608	0.7952	0.7329	0.7144	0.6849	0.6980	0.8090	0.9038
*z* = 1 (*k* = 1)		0.9480	0.8963	0.8447	0.7960	0.7480	0.7387	0.7669	0.8491	0.9050
*z* = 1 (*k* = 2)		0.9447	0.8896	0.8346	0.7819	0.7285	0.7583	0.8284	0.8904	0.9454
*z* = 2 (*k* = 0.5)		0.9309	0.8650	0.8137	0.7758	0.7477	0.7290	0.7647	0.8179	0.9003
*z* = 2 (*k* = 1)		0.9484	0.8973	0.8482	0.8135	0.7882	0.7721	0.7887	0.8451	0.9093
*z* = 2 (*k* = 2)		0.9450	0.8920	0.8381	0.7959	0.7701	0.7566	0.8076	0.8717	0.9358
*z* → ∞ (*k* = 0.5)		0.9302	0.8619	0.8009	0.7490	0.7598	0.7666	0.7926	0.8497	0.9009
*z* → ∞ (*k* = 1)		0.9480	0.8963	0.8459	0.8009	0.7620	0.7798	0.8018	0.8341	0.8859
*z* → ∞ (*k* = 2)		0.9447	0.8895	0.8359	0.7869	0.7444	0.7584	0.7982	0.8812	0.9424

**Table 7:** The satisfaction of different models when *k* = 0.5.

	$\omega_{1}$	0.1	0.2	0.3	0.4	0.5	0.6	0.7	0.8	0.9
Models										
M–V		0.9712	0.8693	0.7674	0.6659	0.5719	0.5050	0.5035	0.6154	0.7731
M–SE		0.9365	0.8826	0.8422	0.8194	0.8153	0.8250	0.8487	0.8826	0.9356
M–FE		0.9055	0.8109	0.7163	0.6218	0.5979	0.6746	0.7556	0.8372	0.9191
M–YE (*z* = 1)		0.9288	0.8614	0.7941	0.7185	0.6542	0.5982	0.5991	0.6773	0.8121
M–YE (*z* = 2)		0.9326	0.8652	0.7978	0.7304	0.6631	0.5957	0.5457	0.6314	0.7750
M–YE (*z* → ∞)		0.8433	0.7821	0.7325	0.6993	0.6792	0.7653	0.7925	0.8200	0.9009
M–PE (*z* = 1)		0.9302	0.8608	0.7952	0.7329	0.7144	0.6849	0.6980	0.8090	0.9038
M–PE (*z* = 2)		0.9309	0.8650	0.8137	0.7758	0.7477	0.7290	0.7647	0.8179	0.9003
M–PE (*z* → ∞)		0.9302	0.8619	0.8009	0.7490	0.7598	0.7666	0.7926	0.8497	0.9009

**Table 8:** The satisfaction of different models when *k* = 1.

	$\omega_{1}$	0.1	0.2	0.3	0.4	0.5	0.6	0.7	0.8	0.9
Models										
M–V		0.9401	0.8727	0.8153	0.7813	0.7871	0.8148	0.8504	0.8918	0.9393
M–SE		0.9516	0.9080	0.8715	0.8453	0.8340	0.8397	0.8583	0.8912	0.9373
M–FE		0.9328	0.8656	0.7983	0.7310	0.6638	0.6784	0.7588	0.8392	0.9196
M–YE (*z* = 1)		0.9458	0.8954	0.8451	0.7948	0.7373	0.6902	0.6444	0.6946	0.8091
M–YE (*z* = 2)		0.9496	0.8992	0.8489	0.7985	0.7481	0.6977	0.6474	0.6567	0.7874
M–YE (*z* → ∞)		0.8625	0.8178	0.7765	0.7440	0.7225	0.7742	0.8014	0.8299	0.9200
M–PE (*z* = 1)		0.9480	0.8963	0.8447	0.7960	0.7480	0.7387	0.7669	0.8491	0.9050
M–PE (*z* = 2)		0.9484	0.8973	0.8482	0.8135	0.7882	0.7721	0.7887	0.8451	0.9093
M–PE (*z* → ∞)		0.9480	0.8963	0.8459	0.8009	0.7620	0.7798	0.8018	0.8341	0.8859

**Table 9:** The satisfaction of different models when *k* = 2.

	$\omega_{1}$	0.1	0.2	0.3	0.4	0.5	0.6	0.7	0.8	0.9
Models										
M–V		0.9358	0.8682	0.8127	0.7809	0.7895	0.8221	0.8646	0.9095	0.9544
M–SE		0.9481	0.9016	0.8626	0.8352	0.8253	0.8357	0.8620	0.9046	0.9520
M–FE		0.9318	0.8635	0.7952	0.7270	0.6587	0.6811	0.7604	0.8397	0.9190
M–YE (*z* = 1)		0.9422	0.8884	0.8345	0.7807	0.7189	0.6732	0.6177	0.6800	0.8404
M–YE (*z* = 2)		0.9461	0.8922	0.8383	0.7844	0.7304	0.6765	0.6226	0.6442	0.7994
M–YE (*z* → ∞)		0.8596	0.8115	0.7665	0.7304	0.7453	0.7451	0.7981	0.8870	0.9424
M–PE (*z* = 1)		0.9447	0.8896	0.8346	0.7819	0.7285	0.7583	0.8284	0.8904	0.9454
M–PE (*z* = 2)		0.9450	0.8920	0.8381	0.7959	0.7701	0.7566	0.8076	0.8717	0.9358
M–PE (*z* → ∞)		0.9447	0.8895	0.8359	0.7869	0.7444	0.7584	0.7982	0.8812	0.9424

**Table 10:** Summary of results from portfolios obtained from all models in this paper ($\omega_{1}$ = 0.5).

Possibilistic Mean		Variance Model (Model 45)			Shannon Entropy Model (Model 49)			Fuzzy Entropy Model (Model 51)		
	Psychological States	*k* = 0.5	*k* = 1	*k* = 2	*k* = 0.5	*k* = 1	*k* = 2	*k* = 0.5	*k* = 1	*k* = 2
Investment Ratio										
*x*_1_ (000651)		0.0641	0.1989	0.2272	0.1355	0.1759	0.2022	0.0500	0.0500	0.0500
*x*_2_ (600340)		0.0500	0.0500	0.0500	0.0890	0.0705	0.0631	0.0500	0.0500	0.0500
*x*_3_ (600887)		0.0500	0.0723	0.0576	0.1046	0.0961	0.0904	0.0500	0.0500	0.0500
*x*_4_ (000858)		0.0527	0.1283	0.1045	0.1376	0.1415	0.1239	0.0500	0.0500	0.0500
*x*_5_ (600196)		0.0500	0.0975	0.0941	0.1070	0.1053	0.1052	0.0500	0.0500	0.0500
*x*_6_ (600297)		0.0500	0.0500	0.0500	0.0759	0.0552	0.0504	0.0500	0.0500	0.0500
*x*_7_ (600977)		0.0500	0.0500	0.0500	0.0728	0.0550	0.0540	0.0500	0.0500	0.0500
*x*_8_ (300015)		0.0851	0.2529	0.2667	0.1530	0.1950	0.2013	0.5000	0.0500	0.0500
*x*_9_ (600339)		0.0500	0.0500	0.0500	0.0549	0.0500	0.0500	0.0500	0.0500	0.0500
*x*_10_ (000166)		0.0500	0.0500	0.0500	0.0698	0.0555	0.0594	0.0500	0.0500	0.0500
Possibilistic mean		0.0018222	0.0054217	0.0056935	0.0041606	0.0048395	0.0049799	0.0056668	0.0012342	0.0010906
Second indicator		0.00002153	0.000199	0.00037026	2.2530	2.1799	2.1590	0.0623	0.0405	0.0536
Satisfaction		0.5719	0.7871	0.7895	0.8422	0.8340	0.8253	0.5979	0.6638	0.6587
*SR*		0.3927	0.3839	0.2959	–	–	–	0.0910	0.0305	0.0104

**Table 11:** Summary of results from portfolios obtained from all models in this paper ($\omega_{1}$ = 0.5) (Continued).

Possibilistic Mean		Yager Entropy Model (Model 53, *z* = 1)			Yager Entropy Model (Model 53, *z* = 2)			Yager Entropy Model (Model 53, *z* → ∞)		
	Psychological States	*k* = 0.5	*k* = 1	*k* = 2	*k* = 0.5	*k* = 1	*k* = 2	*k* = 0.5	*k* = 1	*k* = 2
Investment Ratio										
*x*_1_ (000651)		0.1000	0.1000	0.1000	0.1000	0.1000	0.1000	0.1249	0.1119	0.1517
*x*_2_ (600340)		0.1000	0.1000	0.1000	0.1000	0.1000	0.1000	0.0852	0.0889	0.0638
*x*_3_ (600887)		0.1000	0.1000	0.1000	0.1000	0.1000	0.1000	0.1095	0.1118	0.1103
*x*_4_ (000858)		0.1000	0.1000	0.1000	0.1000	0.1000	0.1000	0.1249	0.1119	0.1517
*x*_5_ (600196)		0.1000	0.1000	0.1000	0.1000	0.1000	0.1000	0.1158	0.1119	0.1154
*x*_6_ (600297)		0.1000	0.1000	0.0979	0.1000	0.1000	0.1000	0.0751	0.0801	0.0593
*x*_7_ (600977)		0.1000	0.1000	0.1000	0.1000	0.1000	0.1000	0.0733	0.0800	0.0603
*x*_8_ (300015)		0.1000	0.1000	0.1000	0.1000	0.1000	0.1000	0.1249	0.1119	0.1517
*x*_9_ (600339)		0.0813	0.0836	0.0852	0.1000	0.1000	0.1000	0.0658	0.0720	0.0569
*x*_10_ (000166)		0.1000	0.0998	0.1000	0.1000	0.1000	0.1000	0.0718	0.0803	0.0622
Possibilistic mean		0.0028059	0.0025533	0.0022618	0.0027078	0.0024685	0.0021813	0.0036976	0.0030996	0.0041239
Second indicator		1.7813	1.7834	1.7831	0.9487	0.9487	0.9487	0.8751	0.8881	0.8483
Satisfaction		0.6542	0.7373	0.7189	0.6631	0.7481	0.6226	0.6792	0.7225	0.7453
*SR*		–	–	–	–	–	–	–	–	–

**Table 12:** Summary of results from portfolios obtained from all models in this paper ($\omega_{1}$ = 0.5) (Continued).

Possibilistic Mean		Proportional Entropy Model (Model 58, *z* = 1)			Proportional Entropy Model (Model 58, *z* = 2)			Proportional Entropy Model (Model 58, *z* → ∞)		
	Psychological States	*k* = 0.5	*k* = 1	*k* = 2	*k* = 0.5	*k* = 1	*k* = 2	*k* = 0.5	*k* = 1	*k* = 2
Investment Ratio										
*x*_1_ (000651)		0.1304	0.1030	0.1016	0.1901	0.1926	0.2108	0.1681	0.1211	0.1242
*x*_2_ (600340)		0.1302	0.1030	0.1016	0.1533	0.1595	0.1614	0.1025	0.1085	0.1105
*x*_3_ (600887)		0.1302	0.1030	0.1016	0.1447	0.1447	0.1403	0.1019	0.1011	0.0976
*x*_4_ (000858)		0.1303	0.1030	0.1016	0.1393	0.1308	0.1233	0.1676	0.1126	0.1103
*x*_5_ (600196)		0.1237	0.1030	0.1016	0.0948	0.0960	0.0896	0.1019	0.0999	0.0966
*x*_6_ (600297)		0.0767	0.0938	0.0933	0.0500	0.0570	0.0500	0.0579	0.0872	0.0829
*x*_7_ (600977)		0.0767	0.0938	0.0933	0.0500	0.0500	0.0500	0.0569	0.0819	0.0833
*x*_8_ (300015)		0.0774	0.0938	0.0933	0.0777	0.0695	0.0745	0.1226	0.0945	0.0970
*x*_9_ (600339)		0.0576	0.0840	0.0872	0.0500	0.0500	0.0500	0.0569	0.0819	0.0833
*x*_10_ (000166)		0.0577	0.0840	0.0872	0.0500	0.0500	0.0500	0.0557	0.0773	0.0789
Possibilistic mean		0.0035896	0.0026054	0.0022611	0.0042043	0.0038709	0.0037876	0.0043993	0.0028775	0.0026252
Second indicator		1.9257	1.9811	1.9856	1.3307	1.3432	1.3300	0.9343	0.9873	0.9863
Satisfaction		0.7144	0.7480	0.7285	0.7477	0.7882	0.7701	0.7598	0.7620	0.7444
*SR*		–	–	–	–	–	–	–	–	–
